# A transcription factor, MrMsn2, in the dimorphic fungus *Metarhizium rileyi* is essential for dimorphism transition, aggravated pigmentation, conidiation and microsclerotia formation

**DOI:** 10.1111/1751-7915.13302

**Published:** 2018-08-29

**Authors:** Zhangyong Song, Jie Yang, Caiyan Xin, Xiaorui Xing, Qing Yuan, Youping Yin, Zhongkang Wang

**Affiliations:** ^1^ School of Basic Medical Sciences Southwest Medical University Luzhou 646000 China; ^2^ Chongqing Engineering Research Center for Fungal Insecticide School of Life Science Chongqing University Chongqing 400030 China

## Abstract

Microsclerotia (MS) are pseudoparenchymatous aggregations of hyphae of fungi that can be induced in liquid culture for biocontrol applications. Previously, we determined that the high‐osmolarity glycerol (HOG) signalling pathway was involved in regulating MS development in the dimorphic insect pathogen *Metarhizium rileyi*. To further investigate the mechanisms by which the signalling pathway is regulated, we characterized the transcriptional factor MrMsn2, a homologue of the yeast C_2_H_2_ transcriptional factor Msn2, which is predicted to function downstream of the HOG pathway in *M. rileyi*. Compared with wild‐type and complemented strains, disruption of *MrMsn2* increased the yeast‐to‐hypha transition rate, enhanced conidiation capacity and aggravated pigmentation in *M. rileyi*. The *▵MrMsn2* mutants were sensitive to stress, produced morphologically abnormal clones and had significantly reduced MS formation and decreased virulence levels. Digital expression profiling revealed that genes involved in antioxidation, pigment biosynthesis and ion transport and storage were regulated by *MrMsn2* during conidia and MS development. Taken together, our findings confirm that MrMsn2 controlled the yeast‐to‐hypha transition, conidia and MS formation, and virulence.

## Introduction

DNA‐binding and multimerization domains are often used to classify transcriptional factors (TFs) into basic leucine zipper, zinc finger motif, helix–turn–helix and helix–loop–helix types (Park *et al*., 2008; Chai *et al*., 2012; Jung *et al*., 2015; Yin *et al*., 2017). As the core of signalling pathway, fungal TFs are important for transcriptional regulation of gene expression during cellular growth, secondary metabolism, stress responses and pathogenesis (Klug, 2010; Hong *et al*., 2013; Liu *et al*., 2013; Marinho *et al*., 2014; Zhang *et al*., 2014; Huang *et al*., 2015; Shelest, 2017; Yin *et al*., 2017; Song *et al*., 2018).

Filamentous fungal Msn2/4 homologues are C_2_H_2_‐like zinc finger TFs that regulate the general stress response, pathogenicity, secondary metabolism and cellular growth. They are similar to ScMsn2/4 of *Saccharomyces cerevisiae* (Schmitt and Mcentee, 1996) and have been characterized in *Aspergillus parasiticus*,* A. flavus*,* Beauveria bassiana*,* Magnaporthe oryzae*,* Metarhizium robertsii* and *Verticillium dahliae* (Chang *et al*., 2011; Liu *et al*., 2013; Zhang *et al*., 2014; Tian *et al*., 2017). Under abiotic and biotic stresses, Msn2/4 is phosphorylated for translocation from the cytoplasm to the nucleus, where it drives the transcription of stress‐induced genes (Hansen *et al*., 2015; Yi and Huh, 2015; Li *et al*., 2017). The underlying mechanism of the regulation of Msn2/4 activity by protein kinase A (PKA), the rapamycin signalling pathway, the Snf1 protein kinase pathway and the high‐osmolarity glycerol (HOG) pathway have been identified (Liu *et al*., 2013; Zhang *et al*., 2014; Li *et al*., 2017).

Microsclerotia (MS) are pseudoparenchymatous aggregations of hyphae with a diameter of 50–600 μm and become melanized during their development. As promising fungal propagules, MS can be induced in liquid culture and used for biocontrol applications such as biofungicides, bioherbicides, bionematicides and mycoinsecticides (Shearer, 2007; Jackson *et al*., 2010; Song *et al*., 2014, 2016a). To enhance the liquid fermentation efficiency of *Metarhizium rileyi* MS, we previously investigated the molecular mechanism of MS formation and demonstrated that internal oxidative stress triggers MS differentiation (Song *et al*., 2013, 2015, 2016b, 2018; Jiang *et al*., 2014). We found that HOG and cell wall integrity (CWI) pathways cooperate to regulate MS formation (Song *et al*., 2016b). We also found that *M. rileyi MrMsn2* was predicted to function downstream of the HOG pathway and was upregulated during MS formation in comparative transcriptome analysis (Song *et al*., 2013). Furthermore, a bioinformatics analysis found no Msn4 orthologues in any public genome databases of *M. rileyi* (Song *et al*., 2013; Shang *et al*., 2016). These results imply a possible involvement of MrMsn2 in the regulation of MS development. However, this function has not been studied clearly.

Moreover, *M. rileyi* is a well‐known dimorphic entomopathogenic fungus with yeast‐like hyphal bodies and a true filamentous growth phase (Boucias *et al*., 2000; Fronza *et al*., 2017), which occurs synchronously *in vivo* and *in vitro* (Pendland and Boucias, 1997; Boucias *et al*., 2016). The yeast‐to‐hypha transition is critical for the pathogenesis and life cycle of dimorphic fungi (Wanchoo *et al*., 2009; Boyce and Adrianopoulos, 2015; Gauthier, 2015; Marcos *et al*., 2016). Although signalling pathways related to dimorphic transition are well characterized in the model yeast *Candida albicans*, the mechanisms are not well defined (Noble *et al*., 2017). Thus, studies on *M. rileyi* are useful model for understanding the dimorphic transition mechanism.

This study seeks to further elucidate the role of MrMsn2 in dimorphism transition, conidiation, virulence and MS formation by phenotypic analyses of deletion/rescue mutants constructed previously (Shao *et al*., 2015; Song *et al*., 2016b). We found that the absence of *MrMsn2* resulted in increased yeast‐to‐hypha transition rate, enhanced conidiation capacity, aggravated pigmentation and induced or suppressed expression of target genes involved in the important phenotypes of *M. rileyi*, as presented below.

## Results

### Molecular characterization of MrMsn2

The full‐length sequence of *MrMsn2* (GenBank Accession No.: MG641237) is 1752 bp, including one intron, with a calculated molecular weight of 56.7 kDa and an isoelectric point of 5.06 (http://expasy.org/tools/protparam.html). Furthermore, MrMsn2 contains a zinc finger double domain (Hu *et al*., 2014). In this study, a phylogenetic tree analysis demonstrated that the MrMsn2 protein from *M. rileyi* was closely related to other *Metarhizium* spp. proteins (Fig. S1). In addition, the amino acid sequence of MrMsn2 showed similarities (79–81% identity) to a cutinase G‐box binding protein of *Metarhizium* spp. (Hu *et al*., 2014; Shang *et al*., 2016) and a zinc finger protein (66% identity) of *Tolypocladium ophiglossoides* (Quandt *et al*., 2015).

To characterize the functions of the MrMsn2 gene in *M. rileyi*, gene replacement mutants and complementary transformants were generated. All recombinant strains were verified by polymerase chain reaction (PCR) and quantitative real‐time PCR (RT–qPCR) screening (Fig. S2). The confirmed *▵MrMsn2* mutants and the complemented (*▵MrMsn2 + *Msn2) strains were used in further experiments.

### MrMsn2 negatively regulates yeast‐to‐hypha transition and conidiation

**Figure 1:**
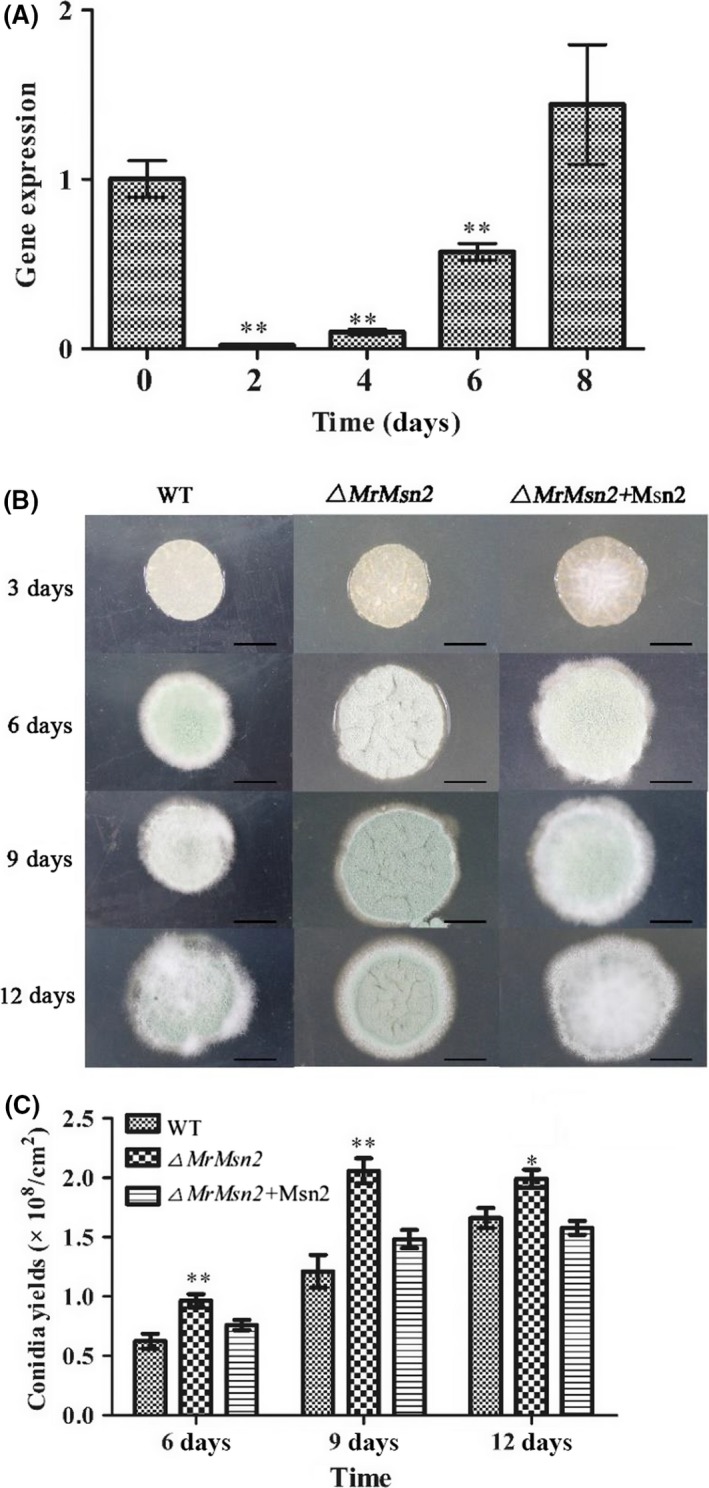
Mycelia growth, conidial morphology and yield of wild‐type, complemented and *▵MrMsn2* mutants strains on SMAY media and RT–qPCR for *MrMsn2* expression during conidiation. A. Transcription of *MrMsn2* during conidiation on different cultivation days. 3 μl of conidial suspensions (1 × 10^7^ conidia ml^−1^) was spotted on SMAY plates and cultured under continuous light at 25°C for 8 days. Stages of conidial development: inoculated conidia at initial culture time (day 0), blastospores (day 2), hyphal period (day 4), conidiation initiation (day 6) and conidia at start of maturation (day 8). B. Images of colonies on SMAY plates for 3, 6, 9 and 12 days. 3 μl of conidial suspensions (1 × 10^7^ conidia ml^−1^) was spotted on SMAY plates and cultured under continuous light at 25°C for 12 days. Bar = 5 mm. C. Conidial yield of tested strains on SMAY medium after 6, 9 and 12 days of incubation. Error bars are standard error. * *P *< 0.05, ** *P *< 0.01, significantly different compared to wild type or 0 day incubation.

The *M. rileyi* CQNr01 (wild‐type, WT) strain was grown on solid Sabouraud maltose agar fortified with yeast extract (SMAY). Compared with the initial results at day 0, expression of *MrMsn2* was found to be downregulated in the yeast‐to‐hypha transition at days 2 and 4 and conidiation initiation at day 6 (Fig. 1A). These results indicate that MrMsn2 may be involved in the yeast‐to‐hypha transition and conidiation.

Further investigations showed that at day 3, the yeast‐to‐hyphae transition was advanced in *▵MrMsn2* mutants compared to the WT and complemented (CP) strains (Fig. 1B). Furthermore, colony surfaces of *▵MrMsn2* mutants were more convoluted compared to the normal smooth colony surfaces of WT and CP strains. After 6 days, the diameter of the mutant colonies was larger compared to that of the WT (Fig. 1B). Additionally, the *▵MrMsn2* mutants had significantly increased conidial yields compared to the WT and CP strains (*P *< 0.001) (Fig. 1C). After 9 and 12 days, conidial yield had increased by 1.5‐ to 2.2‐fold in *▵MrMsn2* mutants compared to the WT and CP strains. Taken together, these transcription and phenotype growth analysis suggest that MrMsn2 is involved in negative control of conidia production and yeast‐to‐hypha transition.

**Figure 2:**
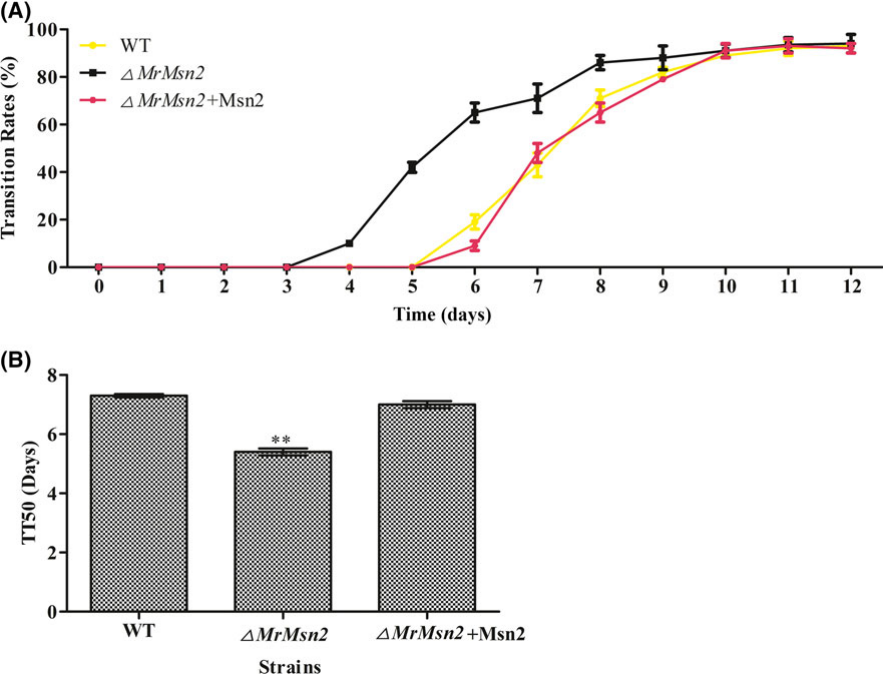
Quantitative analysis dimorphic transition of test strains. A. Quantitative analysis of dimorphic transition (from yeast cells to hypha) rate of wild‐type, complemented and *▵MrMsn2* mutants with approximately 100 single yeast cells plated on SMAY medium. The growth morphology was observed every day. B. Median transition time required for 50% transition of blastospores to hyphae (TT_50_) of wild‐type, complemented and *▵MrMsn2* mutants was compared. TT_50_ was calculated using a probit analysis with the SPSS program. Error bars are standard error. * *P *< 0.05, ** *P *< 0.01, significantly different compared with wild type.

To further analyse the effect of MrMsn2 on the dimorphic transition, yeast cells of test strains was grown on SMAY medium. The investigations into the switching rates and median transition time required for 50% transition of blastospores to hyphae (TT_50_) showed a significantly difference among the *▵MrMsn2* mutants (TT_50_ = 5.4 ± 0.2 days), WT (TT_50_ = 7.3 ± 0.1 days) and CP (TT_50_= 7.0 ± 0.2 days) strains (*P *< 0.001) (Fig. 2). This suggests that deletion of MrMsn2 promoted the yeast‐to‐hypha transition.

### Absence of MrMsn2 leads to aggravated pigmentation

**Figure 3:**
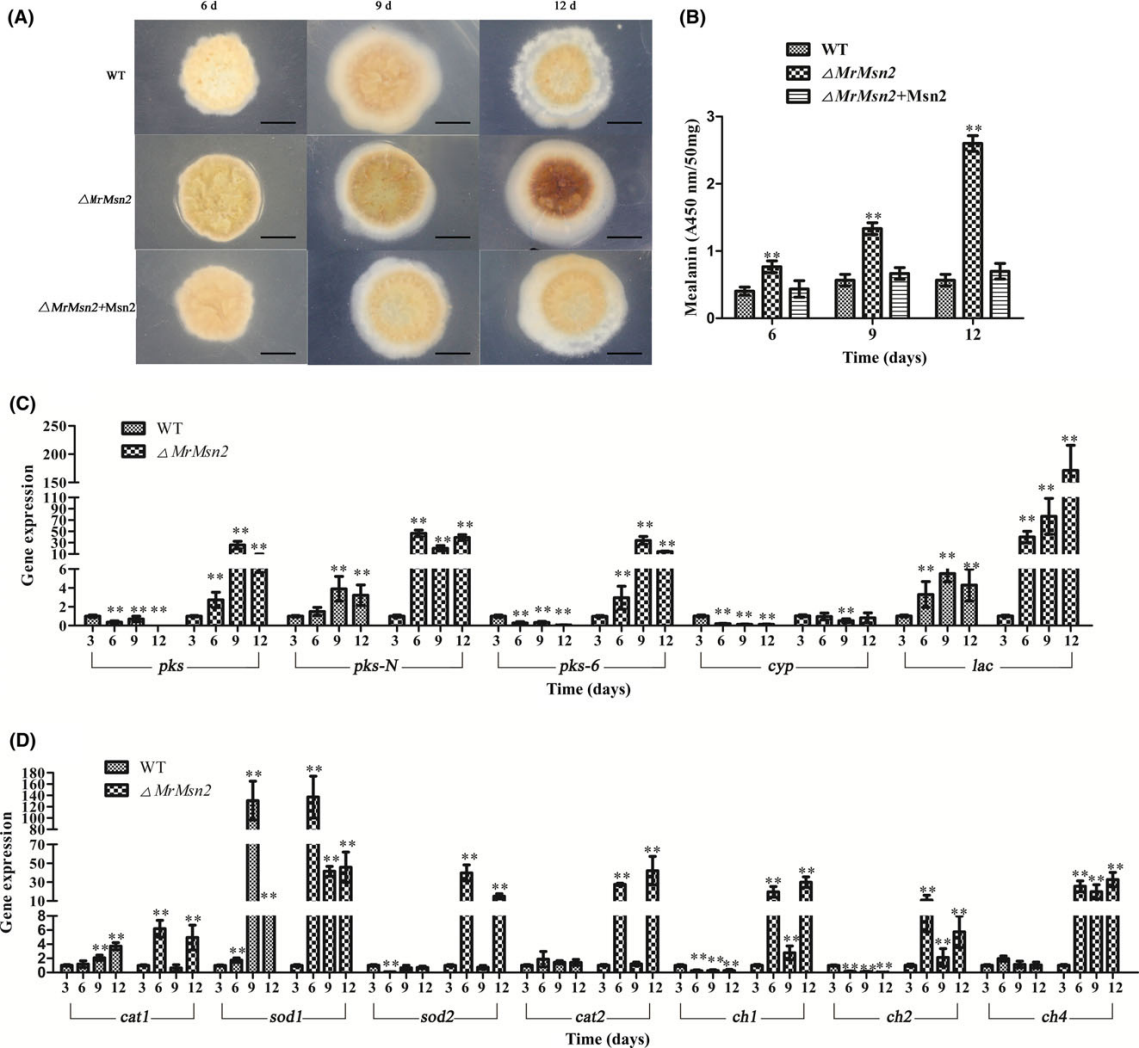
Pigment produced by wild‐type, complemented and *▵MrMsn2* mutant strains and RT–qPCR of pigment biosynthesis, chitin synthase and antioxidant enzyme genes. A. Bottom of morphology clone of wild‐type, omplemented and *▵MrMsn2* mutants. Bar = 5 mm. B. Quantitative analysis pigment produced. Pigment was calculated from three independent experiments and measured spectrophotometrically by absorbance at 459 nm. RT–qPCR of pigment biosynthesis‐related genes (C) and (D) antioxidation genes and chitin synthase genes during conidia development of wild‐type or *▵MrMsn2* mutants. At 3, 6, 9 and 12 days, clones of wild‐type or *▵MrMsn2* mutants were collected for RT–qPCR analysis. *Mrtub* and *Mrtef* genes were used as reference. Error bars are standard error. * *P *< 0.05, ** *P *< 0.01, significantly different compared with the results at day 3.

After incubation on SMAY medium for 6 days, *▵MrMsn2* mutants were found to have altered aggravated pigmentation (Fig. 3A). After 9 and 12 days, the tergal pigment of *▵MrMsn2* mutants had increased compared to WT and CP strains respectively. Furthermore, the pigment concentration in *▵Mrsn2* mutants was found to be significantly increased by factors of 1.8‐ to 4.2‐fold, compared to that of WT and CP strains respectively (Fig. 3B). These data suggest that *MrMsn2* had a negative influence on clone pigment biosynthesis.

To investigate the mechanism of dimorphic transition, pigment biosynthesis and conidiation regulated by *MrMsn2*, genes that were potentially involved were selected from transcriptome libraries (Song *et al*., 2013, 2018) and examined by transcriptional analysis. The following genes were selected: pigment biosynthesis‐related genes (polyketide synthase, *pks*; polyketide synthase–non‐ribosomal peptide synthetase, *pks‐N*; polyketide synthase 6, *pks‐6*; conidial yellow pigment biosynthesis, *cyp*; and laccase, *lac*), several chitin synthase genes (*ch1*,* ch2* and *ch4*, for class I, II and IV chitin synthases respectively) and antioxidant enzyme genes (*cat1* for catalase‐1 and *cat2* for catalase‐2, *sod1* for superoxide dismutase‐1 and *sod2* for superoxide dismutase‐2). It was found that the *lac*,* pks‐N*,* cat1* and *sod1* genes were upregulated, whereas *pks*,* pks‐6*,* cyp*,* ch1* and *ch2* genes were downregulated in the WT strain during conidiation (Figs. 3C and D). Compared with the WT, all pigment biosynthesis‐related gene and chitin synthase genes were significantly upregulated during conidiation in the *▵Mrsn2* mutants (Fig. 3C). In addition, the antioxidant enzyme genes were upregulated in aggravated pigmentation after 6 days for *▵Mrsn2* mutants (Fig. 3D).

### MrMsn2 contributes to tolerance to abiotic stress

To examine the function of MrMsn2 on the abiotic stress response, strains were cultured under various abiotic stress conditions. Convoluted colony surfaces were more apparent, especially under cell wall perturbation and oxidative stress, for *▵MrMsn2* mutants compared to the normal smooth colony surfaces of the WT and CP strains, after 3 day incubation (Fig. S3A). Furthermore, compared to WT strain, smaller colonies were present in the *▵MrMsn2* mutants (Fig. S3A). After 12 days, the conidial yield of *▵MrMsn2* was significantly reduced between 26.6 and 91.9% on SMAY medium, under osmosensitivity, cell wall perturbation or oxidative stress (*P *< 0.001). Interestingly, the conidial yield of *▵MrMsn2* mutants was found to be significantly reduced under KCl stress, however, it was significantly increased under NaCl stress compared to the WT and CP strains (*P *< 0.001) (Fig. S3B). These results indicate *MrMsn2* contributes to tolerance of abiotic stress.

### MrMsn2 is needed for MS development

**Figure 4:**
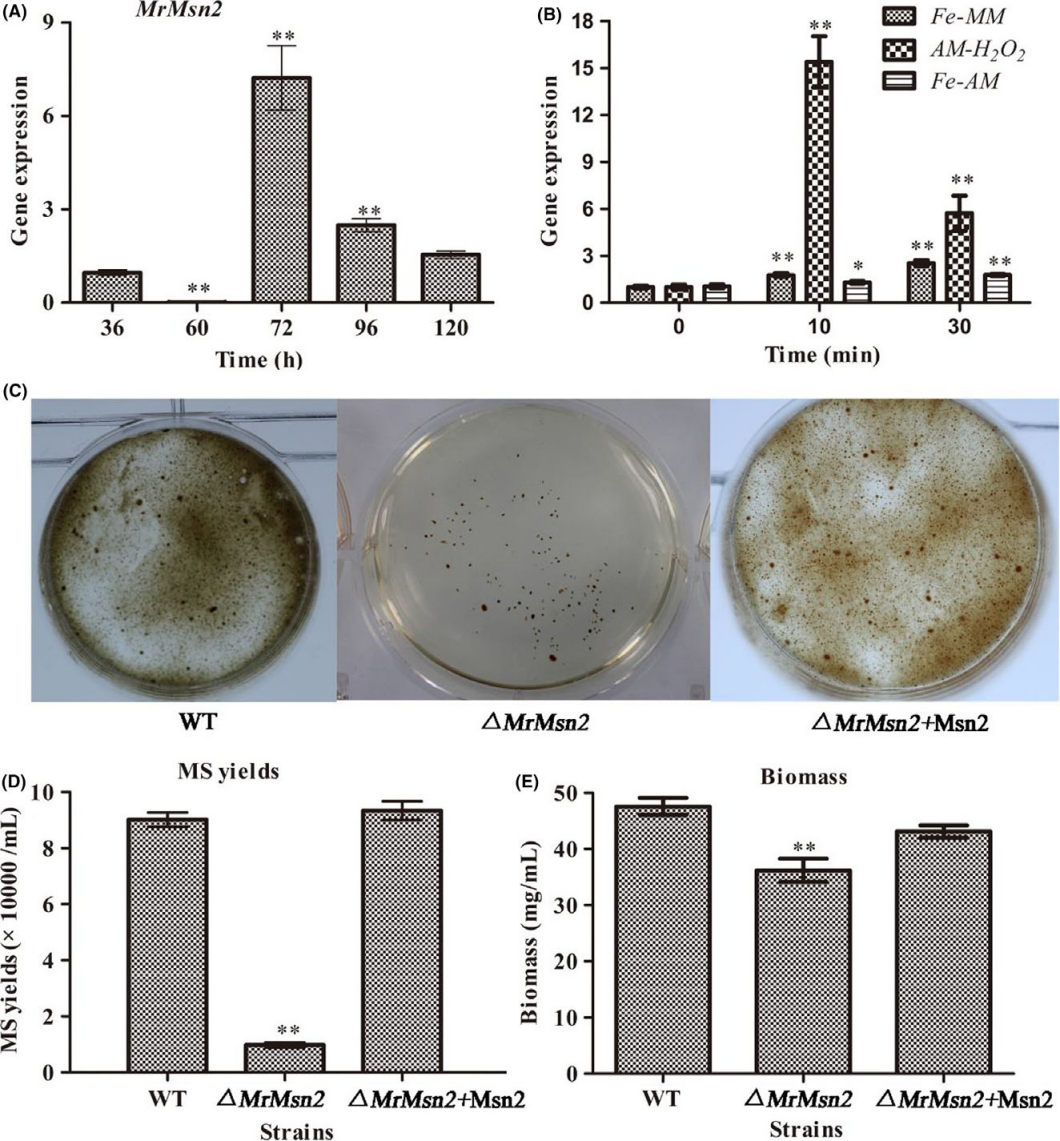
MS development in AM and RT–qPCR of *MrMsn2* during MS development and independent treatment for exogenous oxidative stress. A. Transcription of *MrMsn2* during MS development. B. Relative expression of *MrMsn2* following independent treatments for exogenous iron and oxidative stress. *Mrtub* and *Mrtef* genes were used as reference. C. Phenotypic characterization of MS of tested strains. AM cultures were inoculated with conidial suspensions of tested strains and cultured for 6 days. MS yield (D) and biomass (E) of tested strains. Error bars are standard error. * *P *< 0.05, ** *P *< 0.01, significantly different compared with wild type.

An expression analysis showed that the relative transcriptional of *MrMsn2* peaked with MS initiation (72 h) (Fig. 4A) and *MrMsn2* were upregulated in liquid amended medium (AM) or MM (AM without basal salts) medium, cultured with exogenous oxidative stress (Fig. 4B). These results suggest that *MrMsn2* may be involved in the regulation of MS formation.

After incubation in liquid AM for 144 h, MS produced by WT and CP strains matured and were accompanied by secondary mycelia growth, whereas the density of the induced MS in the *▵MrMsn2* mutants was significantly decreased, with the *▵MrMsn2* culture broth exhibiting a low degree of pigmentation (Fig. 4C). Compared to the WT and CP strains, the MS yield of *▵MrMsn2* mutants was reduced by approximately 88.9% (Fig. 4D), and the biomass was decreased by 23.6% in the AM culture (Fig. 4E). These results indicate that *MrMsn2* is needed for MS development.

### Expression analysis during MS development

**Figure 5:**
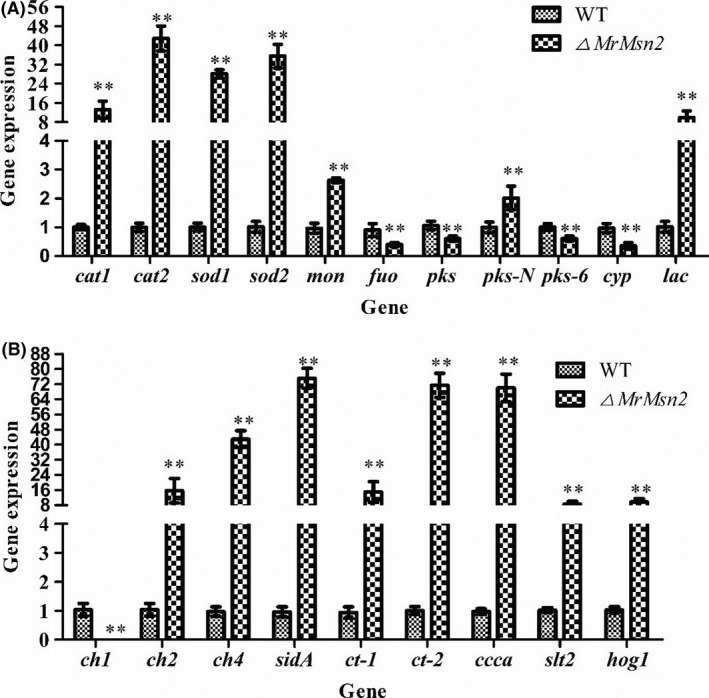
Relative transcripts of MS development‐related genes after 72‐h incubation in AM culture. Transcription of (A) antioxidation genes and pigment biosynthesis‐related genes, (B) chitin synthase genes and major salts transport and store genes, and other genes were carried out during MS development. Wild‐type and *▵MrMsn2* mutants were incubated for 72 h in AM cultures. Relative transcript abundances of genes were measured by RT–qPCR. *Mrtub* and *Mrtef* genes were used as reference. Error bars are standard error. * *P *< 0.05, ** *P *< 0.01, significantly different compared with wild type.

To investigate the genes regulated by *MrMsn2* during MS formation, several groups of genes were analysed by qRT–PCR. It was found that antioxidation genes such as *cat1*,* cat2*,* sod1*,* sod2* and the monooxygenase (*mon*) gene were upregulated in the *▵MrMsn2* mutants, while the flavoprotein–ubiquinone oxidoreductase (*fuo*) gene was downregulated (Fig. 5A). Pigment biosynthesis genes *pks*,* pks‐6* and *cyp* were found to also be downregulated and *pks‐N* and *lac* genes were upregulated in the *▵MrMsn2* mutants (Fig. 5A). Additionally, chitin synthase genes *ch2* and *ch4* were significantly upregulated in the *▵MrMsn2* mutants and *ch1* was significantly downregulated (Fig. 5B). Interestingly, *Slt2*, the core gene of the CWI signalling pathway, and *hog1*, the core gene of the HOG signalling pathway, were significantly upregulated in the *▵MrMsn2* mutants (Fig. 5B). Finally, transport and storage genes for major salts, *sidA* (siderophore iron transporter), *ct‐1* (calcium‐transporting ATPase 1), *ct‐2* (calcium‐transporting p‐type ATPase) and *ccca* (vacuolar Fe^2+^/Mn^2+^ transporter) were all found to be significantly upregulated in the *▵MrMsn2* mutants (Fig. 5B).

### MrMsn2 is required for the virulence of *M. rileyi*

**Figure 6:**
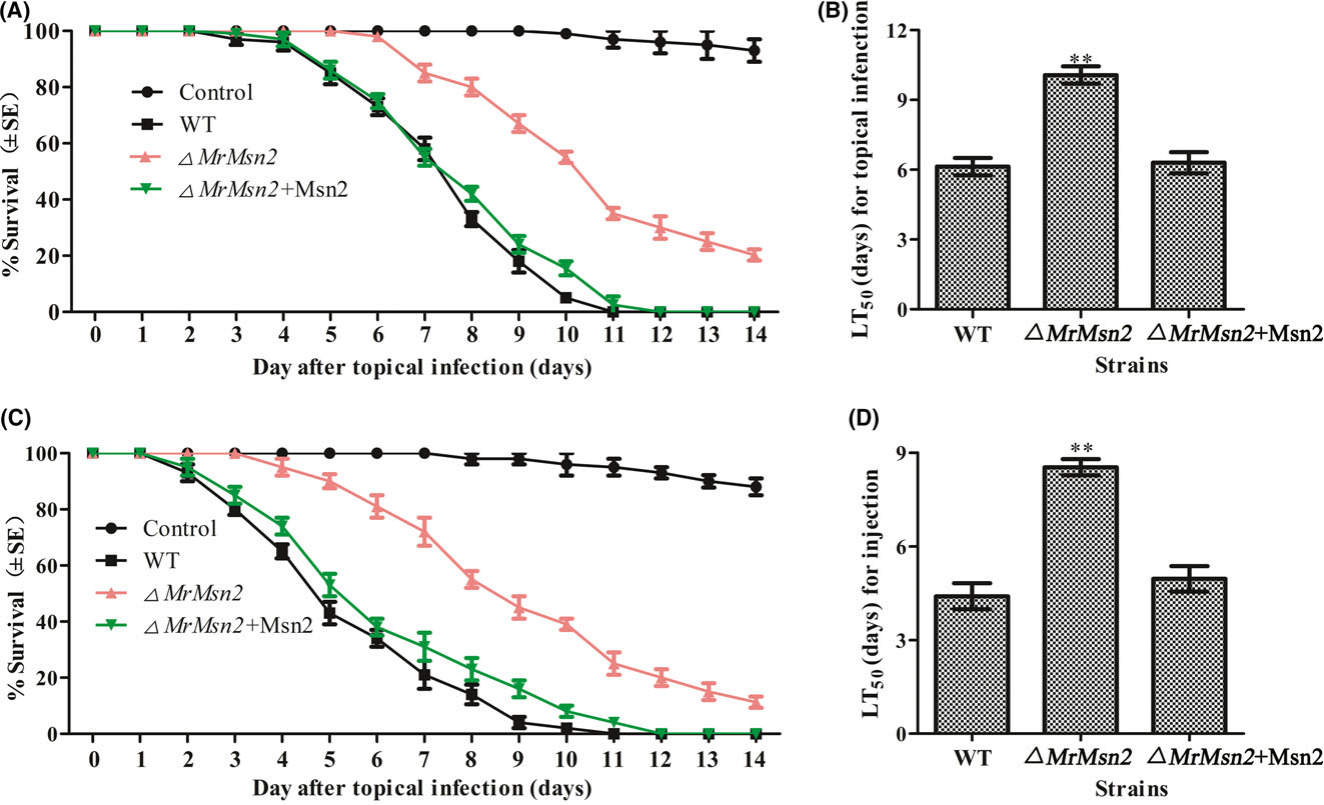
Insect bioassays. Insect survival after (A) topical application or (C) injection of conidia of tested strains. Mean lethal time for (B) topical infections and (D) injection applications. Topical infections were generated by immersing of conidial suspensions of tested strains (5 μl of 1 × 10^7^ conidia ml^−1^ in cotton seed oil). For injection assays, insects were injected conidial suspensions of tested strains (5 μl of 1 × 10^6^ conidia ml^−1^ in sterile water with 0.01% Tween 80). Three replicate groups had 30 larvae each. Controls were treated with pure cotton seed oil or sterile water containing 0.01% Tween 80 only. Error bars are standard error of three trials. * *P *< 0.05, ** *P *< 0.01, significantly different compared with wild type.

Pathogenicity assays were conducted using third‐instar *Spodoptera litura* larvae. These assays showed that the virulence of the *▵MrMsn2* mutants was significantly lower than of the WT and CP strains (Fig. 6). The mean lethal time (LT_50_) for the WT strain was 6.2 ± 0.4 days in a topical bioassay and 4.3 ± 0.5 days in the injection bioassays. The LT_50_ values for the CP strains were 6.3 ± 0.5 days in topical bioassays and 4.9 ± 0.5 days in injection bioassays, whereas the LT_50_ values for the *▵MrMsn2* mutants were 10.1 ± 0.5 (*P *< 0.001) in the topical bioassay and 8.6 ± 0.4 (*P *< 0.001) in the injection bioassays. These results show that *MrMsn2* is required for virulence.

## Discussion

In our previous investigation, we found that the HOG signalling pathway regulated the dimorphic transition and MS development (Song *et al*., 2016b). To better define the mechanisms of regulation, in this study, we identified and characterized the transcription factor *MrMsn2*, which is predicted to be downstream of the HOG pathway in *M. rileyi*. The results were unexpected because the gene was important for conidiation, the yeast‐to‐hypha transition and MS formation.

MrMsn2 belongs to a group of proteins containing C_2_H_2_‐like Zn finger domains that are important in development, secondary metabolism and stress responses (Chang *et al*., 2011; Liu *et al*., 2013; Zhang *et al*., 2014; Tian *et al*., 2017). In this study, *▵MrMsn2* mutants negatively controlled the yeast‐to‐hypha transition (Figs 1 and 2). This result was unlike the observation in *Yarrowia lipolytica* yeast, in which disruption of Mhy1p, an Msn2/4‐like protein, restricted the dimorphic transition, and in *C. albicans* where it had no significant role in CaMsn2/CaMsn4 mutations (Hurtado and Rachubinski, 1999; Nicholls *et al*., 2004). These studies show distinct strategies for regulating the yeast‐to‐hypha transition using Msn2 in different fungi.

Moreover, similar to *A. parasiticus* and *A. flavus* (Chang *et al*., 2011), deletion of *MrMsn2* inhibited growth but increased production of conidia (Fig. 1). This was unlike in *▵MoMsn2* in *M. oryzae*,* ▵Bbmsn2* in *B. bassiana* or *▵Mrmsn2* in *M. robertsii*, which decreased conidia production (Liu *et al.,* 2013; Zhang *et al*., 2014). Msn2‐like proteins do not appear to be important in transcriptional regulation of the stress response in *C. albicans* and *V. dahliae* (Nicholls *et al*., 2004; Tian *et al*., 2017), but are important in the stress response in *C. glabrata*, and *S. cerevisiae* (Schmitt and Mcentee, 1996; Roetzer *et al*., 2008). Our research found that *▵MrMsn2* mutants were defective in response to osmotic, cell wall perturbation and oxidative stress (Fig. S3). These studies show distinct strategies for regulating the stress response and conidiation by Msn2 in different fungi.

In eukaryotes microorganism, cellular developmental processes are reported to correlate with increased reactive oxygen species (ROS) levels (Georgiou *et al*., 2006; Takemoto *et al*., 2007). Fungi have evolved effective antioxidant mechanisms that include enzyme families that act as ROS scavengers. Previous studies have shown members of antioxidant enzymes families such as SODs and CATs may have complementary effects during the cellular developmental processes (Xie *et al*., 2012; Youseff *et al*., 2012; Wang *et al*., 2013; Zhang and Feng, 2018). Our investigations confirmed that antioxidant enzyme genes *cat1*,* sod1*,* cat2* and *sod2* had different expressions during conidia development in *M. rileyi* (Fig. 3). In response to ROS stress, Msn2/4 accumulated in the nucleus, where they promoted transcriptional activation of stress‐responsive genes (Hansen *et al*., 2015; Yi and Huh, 2015). However, deletion of *MrMsn2* increased production of ROS (data not shown) and upregulated expression of antioxidant genes to protect against ROS. Upregulation of these antioxidant genes in *▵MrMsn2* mutants suggests that there may be regulated by other signalling network (Zhang and Feng, 2018).

Secondary metabolism, such as pigments production, is triggered and intensified by ROS build‐up, with pigments being important for protecting the fungi against stress conditions (Cho *et al*., 2012; Hong *et al*., 2013). In the absence of *MrMsn2* aggravated pigmentation and pigment synthesis‐associated genes were significantly upregulated during conidiation in *▵MrMsn2* mutants. Chitin is a main component of cell walls and is related to morphogenesis and adaptation to ecological niches (Roncero, 2002; Liu *et al*., 2017). Expression analysis showed that genes from the class I and II of chitin synthases were downregulated during morphogenesis and conidiation, and then in the absence of *MrMsn2,* these were upregulated (Fig. 3). Based on this data, we propose a link between chitin biosynthesis and MrMsn2, however, the molecular mechanism remains unknown.

Current conidia mass production methods are not cost‐effective, limiting *M. rileyi* commercialization. MS can be used as an alternative fungal propagule for mycoinsecticide (Song *et al*., 2014) and has been used in large‐scale production in submerged fermentation (Song *et al*., 2017). As for solid culture, *▵MrMsn2* mutants were defective in hyphal growth in liquid AM (data not shown). Furthermore, vegetative hyphae are the prerequisite for MS formation (Song *et al*., 2013; Jiang *et al*., 2014). Consistent with the defective MS formation in the *▵MrHog1* mutants, the *▵MrMsn2* mutants had limited ability to form MS (Fig. 4). This result was unlike the observation in *V. dahliae*, in which *VdMsn2* deletion mutants produce more MS than wild type on solid media (Tian *et al*., 2017). Previously, it was found that the CWI and HOG signalling pathways cooperate to regulate MS development (Song *et al*., 2016b). This has been confirmed in this study, with the core genes of the CWI and HOG signalling pathway both being upregulated in the *▵MrMsn2* mutants.

Our previous study demonstrated that intracellular H_2_O_2_ levels fluctuated during MS development and peak at MS initiation stage (Song *et al*., 2018). Antioxidant enzyme genes were significantly upregulated in *▵MrMsn2* mutants (Fig. 5), indicating that intracellular H_2_O_2_ levels were not equilibrated in the MS‐initiating formation. This result confirmed our previous results that oxidative stress triggered MS formation (Song *et al*., 2013, 2015, 2016b, 2018; Jiang *et al*., 2014). As mentioned, the signalling network that regulates the chitin synthesis‐ and pigment synthesis‐associated genes in solid SMAY and liquid AM culture is highly complicated. Major basal salts such as iron and calcium cations are necessary for MS formation (Song *et al*., 2014) and SidA is the major pathway of cellular iron uptake for MS formation (Li *et al*., 2016). We demonstrated that the iron importer and calcium transports, Ct‐1 and Ct‐2, were important in MS formation (Wang and Yin, unpublished data). In addition to similar transcriptional mechanisms by Msn2/4 for regulating *ccc1* in yeast (Li *et al*., 2017), we found multiple transcriptional mechanisms for regulating genes for iron and calcium cation transport and storage by *MrMsn2* during MS development (Fig. 5). However, the transcriptional mechanisms for regulating cation transport are not clear and further experiments are needed to elucidate the multiple mechanisms.

Research on the function of Msn‐like transcriptional factors in pathogenicity is widespread for entomopathogenic, human pathogenic and phytopathogenic fungi (Roetzer *et al*., 2008; Liu *et al*., 2013; Zhang *et al*., 2014). In *M. rileyi*, our data indicated that *▵MrMsn2* mutants were significantly less pathogenic than WT by both topical infection and injection assays (Fig. 6). Similar results were reported for *▵MoMsn2* mutants of *M. oryzae*,* ▵Bbmsn2* mutants of *B. bassiana*, and *▵Mrmsn2* mutants of *M. robertsii* in which gene deletions cause decreased virulence (Liu *et al*., 2013; Zhang *et al*., 2014). In contrast, in *C. glabrata*, Msn2 was found to have no effect on virulence (Roetzer *et al*., 2008). One explanation for this is that *▵MrMsn2* mutants counter oxidative stress from hosts *in vivo* (Song *et al*., 2016a,b) and were hypersensitive to stress. Another explanation involves morphogenic defects in the mutants. This investigation revealed that vegetative growth of *▵MrMsn2* mutants was defective in haemocoel (data not shown). These results could be reasons why the mutants had decreased pathogenicity *in vivo*.

In summary, this study revealed the *MrMsn2* had negative effects on the dimorphic transition and conidiation and was required for abiotic stress resistance, virulence, and MS formation. Furthermore, the current transcriptional networks of *MrMsn2* during conidia and MS development will enhance our ability to comprehensively understand the molecular mechanism of yeast‐to‐hypha transition and conidia and MS development.

## Experimental procedures

### Strains, media and culture conditions

The *M. rileyi* CQNr01 strain was from the Engineering Research Center for Fungal Insecticides, Chongqing, China. WT and engineered strains were cultured on SMAY (Sabouraud maltose agar, fortified with 1% (w/v) yeast extract) under continuous light at 25°C for 12 days for the conidiation assays or in liquid AM (comprising of 40 g l^−1^ glucose, 2.5 g l^−1^ peptone, 5 g l^−1^ yeast extract, 4.0 g l^−1^ KH_2_PO_4_, 0.8 g l^−1^ CaCl_2_.2H_2_O, 0.6 g l^−1^ MgSO_4_.7H_2_O, 0.1 g l^−1^ FeSO_4_.7H_2_O, 37 mg l^−1^ CoCl_2_.6H_2_O, 16 mg l^−1^ MnSO_4_.H_2_O and 14 mg l^−1^ ZnSO_4_.7H_2_O) for the MS incubation assays according to previous methods (Song *et al*., 2016b). *Escherichia coli* DH5α (Invitrogen, Shanghai, China) was used for recombinant DNA manipulation and *Agrobacterium tumefaciens* AGL‐1 (Invitrogen, Shanghai, China) for fungal transformations. Both were cultured as previously described (Shao *et al*., 2015; Song *et al*., 2016b).

### Gene cloning and bioinformatics analyses

To study the potential function of *MrMsn2*, the full genomic DNA sequence of *MrMsn2* was amplified using primers MrMsn2‐L/MrMsn2‐R (Table S1) based on sequences of a previous transcriptomic library (Song *et al*., 2013). The protein sequence and potentially homologues from other fungal species were aligned with Blastp (https://blast.ncbi.nlm.nih.gov/Blast.cgi). Multiple sequences were aligned with DNAMAN software (http://www.lynnon.com). Neighbour‐joining tree was generated using the software MEGA 6.0 (http://www.megasofware.net) (Tamura *et al*., 2013).

### Generation of deletion and complementation mutants

The *M. rileyi* genome was not annotated when we constructed the targeted gene deletion plasmid. Therefore, fusion primer and nested integrated PCR (Wang *et al*., 2011) with primers in Table S1 were used to obtain flanking regions (data not shown). Upstream and downstream flanking sequences were amplified using primers Ms‐LF/Ms‐LR and Ms‐RF/Ms‐RR (Table S1), respectively, digested with restriction endonucleases and inserted into the plasmid pPZP‐Hph‐Knock, a hygromycin B‐resistance vector. The resultant plasmid was named pPZP‐Hph‐*msn2*. For the mutant complementation strains, the open reading frame (ORF) of *MrMsn2* with the promoter and terminator regions, was amplified based on the subsequently public annotated of the *M. rileyi* genome (Shang *et al*., 2016) using the primers Ms‐HF/Ms‐HR (Table S1). PCR products were digested by restriction endonucleases and ligated into the sulfonylurea resistance vector pPZP‐Sur‐Knock to generate the plasmid, pPZP‐Sur‐*msn2*. Disruption and complementation vectors were transformed into *Agrobacterium* and transformants were screened as described previously (Song *et al*., 2018).

### Phenotypic analyses of test strains on SMAY media

To analyse the function of MrMsn2 in yeast‐to‐hyphae transition, vegetative growth, conidial development and abiotic stress tolerance, conidial suspensions of the tested strains were plated on SMAY as previously described (Song *et al*., 2016b). Colony morphology was investigated, and images were collected using a digital camera (60‐mm Macro lens; Canon Inc., Tokyo, Japan) and microscope. Conidia numbers were counted as previously described (Song *et al*., 2015).

In vitro, *M. rileyi* was grown in yeast cell form for 2–4 days on SMAY to transformed into the filamentous form. The switching rates of the tested strains were counted as previously described (Li *et al*., 2016). Approximately 100 simple yeast cells were pipetted onto SMAY medium and grown at 25°C. Switching rates at indicated time points were recorded and TT_50_ was estimated.

### Purification and measurement of clone pigments

Clone pigments of tested strains were counted as described previously (Li *et al*., 2016). Clones from 6‐day‐old, 9‐day‐old or 12‐day‐old SMAY cultures were isolated with 2% NaOH. Collected clones were ground with liquid nitrogen, suspended in NaOH solution and boiled at 100°C for 2 h. Subsequently, solutions were acidified to pH 2.0 with HCl. Precipitates after centrifugation at 6000 × *g* for 15 min, were dissolved in 2% NaOH and measured on a spectrophotometer at the wavelength of 459 nm (Babitskaya *et al*., 2000).

### MS formation assay in liquid AM media

Conidial suspensions of tested strains were inoculated in liquid AM culture for 6 days. Biomass and MS yield were quantified for the AM cultures and determined as previously described (Song *et al*., 2015). MS morphologies were observed using the digital camera.

### Gene expression by qRT–PCR

To assess the effect of exogenous agents on *MrMsn2* expression, AM or MM was supplemented with exogenous 1 M iron or 3 mM H_2_O_2_ as previously described (Song *et al*., 2016b, 2018). Mycelia were subsequently harvested for RNA extraction. For time‐specific expression patterns during conidia development, samples of WT inoculated on SMAY were collected at 0, 2, 4, 6 and 8 days for RNA extraction. To explore the impact on other genes related to dimorphic transition and melanin production during conidiation, WT or *▵MrMsn2* mutants were incubated on SMAY media and 3‐, 6‐, 9‐ or 12‐day‐old clones were collected independently for transcriptional analysis. For time‐specific expression patterns during MS development, samples of WT inoculated in AM were collected at 36, 60, 72, 96 and 120 h for RNA extraction. To investigate the regulation of other genes during MS formation, WT and *▵MrMsn2* mutants were incubated in AM cultures. After 72 h, mycelia were collected and total RNA was prepared. Gene expression patterns were confirmed for samples of WT, *▵MrMsn2* or CP mycelia cultured in AM for 72 h.

Total RNA was collected according to previous methods (Song *et al*., 2015). RT–qPCR was performed using SYBR Green (Invitrogen, Shanghai, China), as per the manufacturer's instructions. β‐tubulin (*Mrtub*) and translation elongation factor (*Mrtef*) genes were used as internal standards. Relative expression levels were evaluated using the 2^−ΔΔCt^ method (Vandesompele *et al*., 2002).

### Insect virulence assays

Topical infections tests and injection assays were conducted as previously described (Song *et al*., 2016b). Three replicate groups had 30 larvae each, and after treatment, the larvae were reared as described previously (Song *et al*., 2015). Larval mortality was recorded daily, and LT_50_ values were calculated using probit analysis with the software SPSS 17.0 (SPSS Inc., Chicago, IL, USA).

### Data analysis

All assays were repeated three times. Data were analysed by one‐way analysis of variance (ANOVA), followed by Duncan's multiple range tests using SPSS 17.0 software. Graphs were constructed in GraphPad Prism 5 software (GraphPad Software Inc., La Jolla, CA, USA). Error bars represent the standard error.

## Conflict of interest

None declared.

## Supporting information


**Fig. S1** Phylogenetic analysis of MrMsn2 protein.
**Fig. S2** Confirmation of gene disruption and complementation.
**Fig. S3** Morphology analysis and conidial yield of wild‐type (WT), *▵MrSwi6* mutants and complemented (CP) strains mutants under abiotic stress.
**Table S1.** Oligonucleotide primers used in this study.

## References

[mbt213302-bib-0001] Babitskaya, V.G. , Shcherba, V.V. , Filimonova, T.V. , and Grigorchuk, E.A. (2000) Melanin pigments from the fungi *Paecilomyces variotii* and *Aspergillus carbonarius* . Appl Biochem Microbiol 36: 128–133. 10780001

[mbt213302-bib-0002] Boucias, D.G. , Tigano, M.S. , Sosa‐Gomez, D.R. , Glare, T.R. , and Inglis, P.W. (2000) Genotypic properties of the entomopathogenic fungus *Nomuraea rileyi* . Biol Control 19: 124–138.

[mbt213302-bib-0003] Boucias, D. , Liu, S. , Meagher, R. , and Baniszewski, J. (2016) Fungal dimorphism in the entomopathogenic fungus *Metarhizium rileyi*: detection of an *in vivo* quorum‐sensing system. J Invertebr Pathol 136: 100–108. 27018146 10.1016/j.jip.2016.03.013

[mbt213302-bib-0004] Boyce, K.J. , and Adrianopoulos, A. (2015) Fungal dimorphism: the switch from hyphae to yeast is a specialized morphogenetic adaption allowing colonization of a host. FEMS Microbiol Rev 39: 797–811. 26253139 10.1093/femsre/fuv035

[mbt213302-bib-0005] Chai, G.H. , Hu, R.B. , Zhang, D.Y. , Qi, G. , Zuo, R. , Cao, Y.P. , *et al* (2012) Comprehensive analysis of CCCH zinc finger family in poplar (*Populus trichocarpa*). BMC Genom 13: 253. 10.1186/1471-2164-13-253 PMC3427045 22708723

[mbt213302-bib-0006] Chang, P.K. , Scharfenstein, L.L. , Luo, M. , Mahoney, N. , Molyneux, R.J. , Yu, J. , *et al* (2011) Loss of *msnA*, a putative stress regulatory gene, in *Aspergillus parasiticus* and *Aspergillus flavus* increased production of conidia, aflatoxins and kojic acid. Toxins 3: 82–104. 22069691 10.3390/toxins3010082 PMC3210457

[mbt213302-bib-0007] Cho, Y. , Srivastava, A. , Ohm, R.A. , Lawrence, C.B. , Wang, K.H. , Grigoriev, I.V. , *et al* (2012) Transcription factor Amr1 induces melanin biosynthesis and suppresses virulence in *Alternaria brassicicola* . PLoS Pathog 8: e1002974. 23133370 10.1371/journal.ppat.1002974 PMC3486909

[mbt213302-bib-0008] Fronza, E. , Specht, A. , Heinzen, H. , and de Barros, N.M. (2017) *Metarhizium (Nomuraea) rileyi* as biological control agent. Biocontrol Sci Techn 2: 1–22.

[mbt213302-bib-0009] Gauthier, G.M. (2015) Dimorphism in fungal pathogens of mammals, plants, and insects. PLoS Pathog 11: e1004608. 25675433 10.1371/journal.ppat.1004608 PMC4335504

[mbt213302-bib-0010] Georgiou, C.D. , Patsoukis, Ν. , Papapostolou, Ι. , and Zervoudakis, G. (2006) Sclerotial metamorphosis in filamentous fungi is induced by oxidative stress. Integr Comp Biol 46: 691–712. 21672779 10.1093/icb/icj034

[mbt213302-bib-0011] Hansen, A.S. , Hao, N. , and O'Shea, E.K. (2015) High‐throughput microfluidics to control and measure signaling dynamics in single yeast cells. Nat Protoc 10: 1181–1197. 26158443 10.1038/nprot.2015.079 PMC4593625

[mbt213302-bib-0012] Hong, S.Y. , Roze, L.V. , and Linz, J.E. (2013) Oxidative stress‐related transcription factors in the regulation of secondary metabolism. Toxins 5: 683–702. 23598564 10.3390/toxins5040683 PMC3705287

[mbt213302-bib-0013] Hu, X. , Xiao, G.H. , Zheng, P. , Shang, Y.F. , Su, Y. , Zhang, X.Y. , *et al* (2014) Trajectory and genomic determinants of fungal‐pathogen speciation and host adaptation. Proc Natl Acad Sci USA 111: 16796–16801. 25368161 10.1073/pnas.1412662111 PMC4250126

[mbt213302-bib-0014] Huang, W. , Shang, Y.F. , Chen, P.L. , Cen, K. , and Wang, C.S. (2015) Basic leucine zipper (bZIP) domain transcription factor MBZ1 regulates cell wall integrity, spore adherence, and virulence in *Metarhizium robertsii* . J Biol Chem 290: 8218–8231. 25673695 10.1074/jbc.M114.630939 PMC4375478

[mbt213302-bib-0015] Hurtado, C.A. , and Rachubinski, R.A. (1999) MHY1 encodes a C2H2‐type zinc finger protein that promotes dimorphic transition in the yeast *Yarrowia lipolytica* . J Bacteriol 181: 3051–3057. 10322005 10.1128/jb.181.10.3051-3057.1999 PMC93759

[mbt213302-bib-0016] Jackson, M.A. , Dunlap, C.A. , and Jaronski, S.T. (2010) Ecological considerations in producing and formulating fungal entomopathogens for use in insect biocontrol. Biocontrol 55: 129–145.

[mbt213302-bib-0017] Jiang, S.S. , Yin, Y.P. , Song, Z.Y. , Zhou, G.L. , and Wang, Z.K. (2014) RacA and Cdc42 regulate polarized growth and microsclerotium formation in the dimorphic fungus *Nomuraea rileyi* . Res Microbiol 165: 233–242. 24657749 10.1016/j.resmic.2014.03.003

[mbt213302-bib-0018] Jung, K.O. , Yang, D.H. , Maeng, S. , Lee, K.T. , So, Y.S. , Hong, J. , *et al* (2015) Systematic functional profiling of transcription factor networks in *Cryptococcus neoformans* . Nat Commun 6: 6757. 25849373 10.1038/ncomms7757 PMC4391232

[mbt213302-bib-0019] Klug, A. (2010) The discovery of zinc fingers and their development for practical application. Annu Rev Biochem 43: 213–231. 10.1146/annurev-biochem-010909-095056 20192761

[mbt213302-bib-0020] Li, Y. , Wang, Z.K. , Liu, X.E. , Song, Z.Y. , Li, R. , Shao, C.W. , *et al* (2016) Siderophore biosynthesis but not reductive iron assimilation is essential for the dimorphic fungus *Nomuraea rileyi* conidiation, dimorphism transition, resistance to oxidative stress, pigmented microsclertium formation, and virulence. Front Microbiol 7: 931. 27379061 10.3389/fmicb.2016.00931 PMC4909778

[mbt213302-bib-0021] Li, L. , Kaplan, J. , and Ward, D.M. (2017) The glucose sensor Snf1 and the transcription factors Msn2 and Msn4 regulate transcription of the vacuolar iron importer gene CCC1 and iron resistance in yeast. J Biol Chem 292: 15577–15586. 28760824 10.1074/jbc.M117.802504 PMC5602413

[mbt213302-bib-0022] Liu, Q. , Ying, S.H. , Li, J.G. , Tian, C.G. , and Feng, M.G. (2013) Insight into the transcriptional regulation of Msn2 required for conidiation, multi‐stress responses and virulence of two entomopathogenic fungi. Fungal Genet Biol 54: 42–51. 23466345 10.1016/j.fgb.2013.02.008

[mbt213302-bib-0023] Liu, R. , Xu, C. , Zhang, Q.Q. , Wang, S.Y. , and Fang, W.G. (2017) Evolution of the chitin synthase gene family correlates with fungal morphogenesis and adaption to ecological niches. Sci Rep 7: 44527. 28300148 10.1038/srep44527 PMC5353729

[mbt213302-bib-0024] Marcos, C.M. , de Oliveira, H.C. , de Melo, W.M. , da J.S., A. , P.A., S. and L., etal. (2016) Anti‐immune strategies of pathogenic fungi. Front Cell Infect Microbiol 6, 142. 27896220 10.3389/fcimb.2016.00142 PMC5108756

[mbt213302-bib-0025] Marinho, H.S. , Real, C. , Cyrne, L. , Soares, H. , and Antunes, F. (2014) Hydrogen peroxide sensing, signaling and regulation of transcription factors. Redox Biol 2: 535–562. 24634836 10.1016/j.redox.2014.02.006 PMC3953959

[mbt213302-bib-0026] Nicholls, S. , Straffon, M. , Enjalbert, B. , Nantel, A. , Macaskill, S. , Whiteway, M. , *et al* (2004) Msn2‐and Msn4‐like transcription factors play no obvious roles in the stress responses of the fungal pathogen *Candida albicans* . Eukaryot Cell 3: 1111–1123. 15470239 10.1128/EC.3.5.1111-1123.2004 PMC522590

[mbt213302-bib-0027] Noble, S.M. , Gianetti, B.A. , and Witchley, J.N. (2017) *Candida albicans* cell‐type switching and functional plasticity in the mammalian host. Nat Rev Microbiol 15: 96. 27867199 10.1038/nrmicro.2016.157 PMC5957277

[mbt213302-bib-0028] Park, J. , Park, J. , Jang, S. , Kim, S. , Kong, S. , Choi, J. , *et al* (2008) FTFD: an informatics pipeline supporting phylogenomic analysis of fungal transcription factors. Bioinformatics 24: 1024–1025. 18304934 10.1093/bioinformatics/btn058

[mbt213302-bib-0029] Pendland, J.C. , and Boucias, D.G. (1997) *In vitro* growth of the entomopathogenic hyphomycete *Nomuraea rileyi* . Mycologia 89: 66–71.

[mbt213302-bib-0030] Quandt, C.A. , Bushley, K.E. , and Spatafora, J.W. (2015) The genome of the truffle‐parasite *Tolypocladium ophioglossoides* and the evolution of antifungal peptaibiotics. BMC Genom 16: 553. 10.1186/s12864-015-1777-9 PMC4517408 26215153

[mbt213302-bib-0031] Roetzer, A. , Gregori, C. , Jennings, A.M. , Quintin, J. , Ferrandon, D. , Butler, G. , *et al* (2008) *Candida glabrata* environmental stress response involves *Saccharomyces cerevisiae* Msn2/4 orthologous transcription factors. Mol Microbiol 69: 603–620. 18547390 10.1111/j.1365-2958.2008.06301.x PMC2610386

[mbt213302-bib-0032] Roncero, C. (2002) The genetic complexity of chitin synthesis in fungi. Curr Genet 41: 367–378. 12228806 10.1007/s00294-002-0318-7

[mbt213302-bib-0033] Schmitt, A.P. , and Mcentee, K. (1996) Msn2p, a zinc finger DNA‐binding protein, is the transcriptional activator of the multistress response in *Saccharmyces cerevisiae* . Proc Natl Acad Sci USA 93: 5777–5782. 8650168 10.1073/pnas.93.12.5777 PMC39137

[mbt213302-bib-0034] Shang, Y.F. , Xiao, G.H. , Zheng, P. , Cen, K. , Zhan, S. , and Wang, C.S. (2016) Divergent and convergent evolution of fungal pathogenicity. Genome Biol Evol 8: 1374–1387. 27071652 10.1093/gbe/evw082 PMC4898799

[mbt213302-bib-0035] Shao, C.W. , Yin, Y.P. , Qi, Z.R. , Li, R. , Song, Z.Y. , and Wang, Z.K. (2015) *Agrobacterium tumefaciens* mediated transformation of the entomopathogenic fungus *Nomuraea rileyi* . Fungal Genet Biol 83: 19–25. 26275508 10.1016/j.fgb.2015.08.002

[mbt213302-bib-0036] Shearer, J.F. (2007) Some observations concerning microsclerotia and spore production of *Mycoleptodiscus terrestris* in culture. Mycologia 99: 88–90. 17663126 10.3852/mycologia.99.1.88

[mbt213302-bib-0037] Shelest, E. (2017) Transcription factors in fungi: TFome dynamics, three major families, and dual‐specificity TFs. Front Genet 8: 53. 28523015 10.3389/fgene.2017.00053 PMC5415576

[mbt213302-bib-0038] Song, Z.Y. , Yin, Y.P. , Jiang, S.S. , Liu, J.J. , Chen, H. , and Wang, Z.K. (2013) Comparative transcriptome analysis of microsclerotia development in *Nomuraea rileyi* . BMC Genom 14: 411. 10.1186/1471-2164-14-411 PMC3698084 23777366

[mbt213302-bib-0039] Song, Z.Y. , Yin, Y.P. , Jiang, S.S. , Liu, J.J. , and Wang, Z.K. (2014) Optimization of culture medium for microsclerotia production by *Nomuraea rileyi* and analysis of their viability for use as a mycoinsecticide. Biocontrol 59: 597–605.

[mbt213302-bib-0040] Song, Z.Y. , Shen, L. , Yin, Y.P. , Tan, W.Y. , Shao, C.W. , Xu, J.M. , *et al* (2015) Role of two *Nomuraea rileyi* transmembrane sensors Sho1p and Sln1p in adaptation to stress due to changing culture conditions during microsclerotia development. World J Microb Biot 31: 477–485. 10.1007/s11274-015-1801-x 25595731

[mbt213302-bib-0041] Song, Z.Y. , Shen, L. , Zhong, Q. , Yin, Y.P. , and Wang, Z.K. (2016a) Liquid culture production of microsclerotia of *Purpureocillium lilacinum* for use as bionematicide. Nematology 18: 719–726.

[mbt213302-bib-0042] Song, Z.Y. , Zhong, Q. , Yin, Y.P. , Shen, L. , Li, Y. , and Wang, Z.K. (2016b) The high osmotic response and cell wall integrity pathways cooperate to regulate morphology, microsclerotia development, and virulence in *Metarhizium rileyi* . Sci Rep 6: 38765. 27941838 10.1038/srep38765 PMC5150533

[mbt213302-bib-0043] Song, Z.Y. , Lin, Y.L. , Du, F. , Yin, Y.P. , and Wang, Z.K. (2017) Statistical optimisation of process variables and large scale production of *Metarhizium rileyi* (Ascomycetes: Hypocreales) microsclerotia in submerged fermentation. Mycology 8: 39–47.

[mbt213302-bib-0044] Song, Z.Y. , Yin, Y.P. , Lin, Y.L. , Du, F. , Ren, G.W. , and Wang, Z.K. (2018) The bZip transcriptional factor activator protein‐1 regulates *Metarhizium rileyi* morphology and mediates microsclerotia formation. Appl Microbiol Biot 102: 4577–4588. 10.1007/s00253-018-8941-5 29589093

[mbt213302-bib-0045] Takemoto, D. , Tanaka, A. , and Scott, B. (2007) NADPH oxidases in fungi: diverse roles of reactive oxygen species in fungal cellular differentiation. Fungal Genet Biol 44: 1065–1076. 17560148 10.1016/j.fgb.2007.04.011

[mbt213302-bib-0046] Tamura, K. , Stecher, G. , Peterson, D. , Filipski, A. , and Kumar, S. (2013) MEGA6: molecular evolutionary genetics analysis version 6.0. Mol Biol Evol 30: 2725–2729. 24132122 10.1093/molbev/mst197 PMC3840312

[mbt213302-bib-0047] Tian, L. , Yu, J. , Wang, Y. , and Tian, C. (2017) The C2H2 transcription factor *VdMsn2* controls hyphal growth, microsclerotia formation, and virulence of *Verticillium dahlia* . Fungal Biol 121: 1001–1010. 29122172 10.1016/j.funbio.2017.08.005

[mbt213302-bib-0048] Vandesompele, J. , De Preter, K. , Pattyn, F. , Poppe, B. , Roy, N.V. , Paepe, A.D. , *et al* (2002) Accurate normalization of real‐time quantitative RT‐PCR data by geometric averaging of multiple internal control genes. Genome Biol 3, Research 0034. 10.1186/gb-2002-3-7-research0034 PMC126239 12184808

[mbt213302-bib-0049] Wanchoo, A. , Lewis, M.W. , and Keyhani, N.O. (2009) Lection mapping reveals stage‐specific display of surface carbohydrates in *in vitro* and haemolymph‐derived cell of the entomopathogenic fungus *Beauveria bassiana* . Microbiology 155: 3131–3133. 10.1099/mic.0.029157-0 19608611

[mbt213302-bib-0050] Wang, Z. , Ye, S.F. , Li, J.J. , Zheng, B. , Bao, M.Z. , and Ning, G.G. (2011) Fusion primer and nested integrated PCR (*FPNI‐PCR*): a new high‐efficiency strategy for rapid chromosome walking or flanking sequence cloning. BMC Biotechnol 11: 109. 22093809 10.1186/1472-6750-11-109 PMC3239319

[mbt213302-bib-0051] Wang, Z.L. , Zhang, L.B. , Ying, S.H. , and Feng, M.G. (2013) Catalases play differentiated roles in the adaptation of a fungal entomopathogen to environmental stress. Environ Microbiol 15: 409–418. 22891860 10.1111/j.1462-2920.2012.02848.x

[mbt213302-bib-0052] Xie, X.Q. , Li, F. , Ying, S.H. , and Feng, M.G. (2012) Additive contributions of two manganese‐cored superoxide dismutases (MnSODs) to antioxidation, UV tolerance and virulence of *Beauveria bassiana* . PLoS ONE 7: e30298. 22279579 10.1371/journal.pone.0030298 PMC3261187

[mbt213302-bib-0053] Yi, D.G. , and Huh, W.K. (2015) PKA, PHO and stress response pathways regulate the expression of UDP‐glucose pyrophosphorylase through Msn2/4 in budding yeast. FEBS Lett 589: 2409–2416. 26188548 10.1016/j.febslet.2015.07.015

[mbt213302-bib-0054] Yin, W.X. , Cui, P. , Wei, W. , Lin, Y. , and Luo, C.X. (2017) Genome‐wide identification and analysis of the basic leucine zipper (bZIP) transcription factor gene family in *Ustilaginoides virens* . Genome 60: 1051–1059. 28841402 10.1139/gen-2017-0089

[mbt213302-bib-0055] Youseff, B.H. , Holbrook, E.D. , Smolnycki, K.A. , and Rappleye, C.A. (2012) Extracellular superoxide dismutase protects histoplasma yeast cells from host‐derived oxidative stress. PLoS Pathog 8: e1002713. 22615571 10.1371/journal.ppat.1002713 PMC3355102

[mbt213302-bib-0056] Zhang, L.B. and Feng, M.G. (2018) Antioxidant enzymes and their contributions to biological control potential of fungal insect pathogens. Appl Microbiol Biot 102, 4995–5004. 10.1007/s00253-018-9033-2 29704043

[mbt213302-bib-0057] Zhang, H. , Zhao, Q. , Guo, X. , Guo, M. , Qi, Z. , Tang, W. , *et al* (2014) Pleiotropic function of the putative zinc‐finger protein MoMsn2 in *Magnaporthe oryzae* . Mol Plant Microbe Interact 27: 446–460. 24405033 10.1094/MPMI-09-13-0271-R

