# Supplementary material for: A transcription factor, MrMsn2, in the dimorphic fungus Metarhizium rileyi is essential for dimorphism transition, aggravated pigmentation, conidiation and microsclerotia formation
Source: Microb Biotechnol. 2018 Aug 29;11(6):1157–69. doi: 10.1111/1751-7915.13302 (PMC6196401; doi:10.1111/1751-7915.13302)
Supplement: Supplementary file 1 — Fig. S1 Phylogenetic analysis of MrMsn2 protein. Fig. S2 Confirmation of gene disruption and complementation. Fig. S3 Morphology analysis and conidial yield of wild‐type (WT), ▵MrSwi6 mutants and complemented (CP) strains mutants under abiotic stress. Table S1. Oligonucleotide primers used in this study. [file MBT2-11-1157-s001.docx]

Table S1 Oligonucleotide primers used in this study

| Usage | Primers | Sequences (5’ to 3’) |
| --- | --- | --- |
| PCR of genomic DNA sequences | MrMsn2-F | GTCTGCCGTCCATTCATTGTC |
|  | MrMsn2-R | CTGACCAAGCACACGATAACG |
| FPNI-PCR of the up- and down-stream genomic DNA flanking sequence | universal primers used for FPNI-PCR | In Reference (Song *et al.,* 2016) |
|  | MrMsn2-1F1 | GAGGGCTGTGTAAATGAGAGGACTGG |
|  | MrMsn2-1F2 | GAGGTCACAAGAAGGGAAAAGAAGGA |
|  | MrMsn2-1F3 | GTGGTGACGGGGTCCATAGGCATAA |
|  | MrMsn2-1R1 | TGCTCGGGATGTTTAGGAAGACAAG |
|  | MrMsn2-1R2 | TGGACATGACTGGGAATCAGTAGGG |
|  | MrMsn2-1R3 | GTCACGACTCCCACGGTCACATCCT |
|  | MrMsn2-2F1 | CATACGTTGCGAAGGGCAGGAGGGC |
|  | MrMsn2-2F2 | GTGGCATTACTCGGGTCAATGGAT |
|  | MrMsn2-2F3 | CGCCCTTTTTTTGGCTTTGCGTGAC |
|  | MrMsn2-2R1 | AAGAAGACGCCTTCCCGACCACTGG |
|  | MrMsn2-2R2 | CGGGTGATGCGGGTTTCCGTTTACT |
|  | MrMsn2-2R3 | TGACGGAACGGCGGTGTCTTCGGTA |
| Construction of gene disruption vector | MsLF | cgGAATTCAGCCAAAAAAAGGGCGTCGGTCACT  *Eco*RI site is underlined |
|  | MsLR | ccgCTCGAGTCGGAAATATGGTCAAAAGCAAAG  *Xho*I site is underlined |
|  | MsRF | gcTCTAGATATCTCATCAATGCGGTTTCCTCGGC  *Xba*I site is underlined |
|  | MsRR | aaCTGCAGCGCACGCATATAGTTACCAACCATCT  *Pst*I site is underlined |
| Construction of gene complementation | Ms-HF | ccgCTCGAGCACGCACGCCTCTCCAGTCTCATCA  *Xho*I site is underlined |
|  | Ms-HR | cccAAGCTTACCTGACCAAGCACACGATAACGAA  *Hin*dIII site is underlined |
| RT-PCR verification | MsF | TTGGTCATTGAGAGGGATTAGTAGT |
|  | MsR | CGGGGCTCCTCCGTCAATACACAAC |
|  | Ms-OF | TTCTGGCAGTGCTGTTGGCAGTCCC |
|  | Ms-OR | GGCGTGGTCATCTACCGATGGAGGA |
|  | hph-F | GCTCTCGCTAAACTCCCCAATGTCA |
|  | hph-R | CATTGACTGGAGCGAGGCGATGTTC |
| For RT-qPCR analysis | Mrmsn2-qF | CAACCCAAGTCTTTCCGATT |
|  | Mrmsn2-qR | CAGGGCTGAAGTCGAATGTA |
|  | Mrtef-qF | GTCATCGTCCTCAACCATC |
|  | Mrtef-qR | CAGTCTCAACAGCCTTACC |
|  | Mrtub-qF | GGCAAGGTCGCTATGAAG |
|  | Mrtub-qR | CTGGATGGAGGTAGAGTTAC |
|  | Mrpks-qF | GCCTCCTCCATCGTCAAGGT |
|  | Mrpks-qR | GCGCTCGTCTCGAGGAAGTA |
|  | Mrpks-N-qF | AGAGCTCGTTGACCCAGTCG |
|  | Mrpks-N-qR | TGGCCTCGACGTGTGTATCC |
|  | Mrpks6-qF | GGACGGTTCAAGCACACGAC |
|  | Mrpks6-qR | TGCAACCGCCACGAGTATCT |
|  | Mrcyp-qF | CCTGTCATATCCGGAGCAGT |
|  | Mrcyp-qR | AGGCCAGGTGCTTTGTAGAT |
|  | Mrcat1-qF | GAGCCAGCCTCGTCTGTTCT |
|  | Mrcat1-qR | CTGGGCGAGGACGTTCTTCT |
|  | Mrsod1-qF | TGCCTGCTTTATCAATCGAC |
|  | Mrsod1-qR | TCAAGAATGAGTGTGCGTCA |
|  | Mrsod2-qF | AGGTTCTCCCAGAAGAGGGA |
|  | Mrsod2-qR | CGTACGTGACGAACCTCAAC |
|  | Mrlac-qF | AGTGCACCGCAAACAATCAT |
|  | Mrlac-qR | GCGACTGTCTGGTTGAAGAC |
|  | Mrcat2-qF | AAGGATCCGCAGAAGCGTGA |
|  | Mrcat2-qR | AAGGTGTGGCCTCCAGCAAT |
|  | Mrchs1-qF | CATCCGTCAACACCAAAGAC |
|  | Mrchs1-qR | GCTGAATAAGGCGACCTCTC |
|  | Mrchs2-qF | AATCGGCGACAATTTCTACC |
|  | Mrchs2-qR | ATTCGTATCCTGCCTTCCAC |
|  | Mrchs4-qF | GTCAATGAGCTCGAAAGTCG |
|  | Mrchs4-qR | TCGACGTCATCTACAAAGGC |
|  | Mrhog1-qF | GCATCGTGACTTGAAGCCTA |
|  | Mrhog1-qR | GGGCTCGGTAATATCGTGTT |
|  | Mrslt2-qF | TGTGCGGCCTCAAGTATATC |
|  | Mrslt2-qR | CGAGGCCAAAGTCACAGAT |
|  | Mrfuo-qF | GTCCCTTGTCGAACTTTCCG |
|  | Mrfuo-qR | CATGGAGTTTCACGCCAGAG |
|  | MrsidA-qF | ACGTATCGGCGATGAAGACT |
|  | MrsidA-qR | CGCTGGTGGTCTTCTTCTTG |
|  | Mrccca-qF | AACAGAGCGCGATACATACG |
|  | Mrccca-qR | GTCGTAGCGTTCGAGGATG |
|  | Mrct-1-qF | GCGCTACCGGCATTGTCATC |
|  | Mrct-1-qR | AGGGCCTTGGACACAGCATT |
|  | Mrct-2-qF | CCATTGTTGATCCGCTGAGG |
|  | Mrct-2-qR | CAAAGCGAAGTCGCCAGTAA |

**Reference**

1. Song, Z.Y., Zhong, Q., Yin, Y.P., Shen, L., Li, Y., and Wang, Z.K. (2016) The high osmotic response and cell wall integrity pathways cooperate to regulate morphology, microsclerotia development, and virulence in *Metarhizium rileyi*. *Sci Rep* **6**: 38765.

**Figure legends**

**Fig. S1** Phylogenetic analysis of MrMsn2 protein.

**Fig. S2** Confirmation of gene disruption and complementation.

**Fig. S3** Morphology analysis and conidial yield of wild-type (WT), *△MrSwi6* mutants and complemented (CP) strains mutants under abiotic stress.

**Fig. S1** Phylogenetic tree analysis MrMsn2 protein
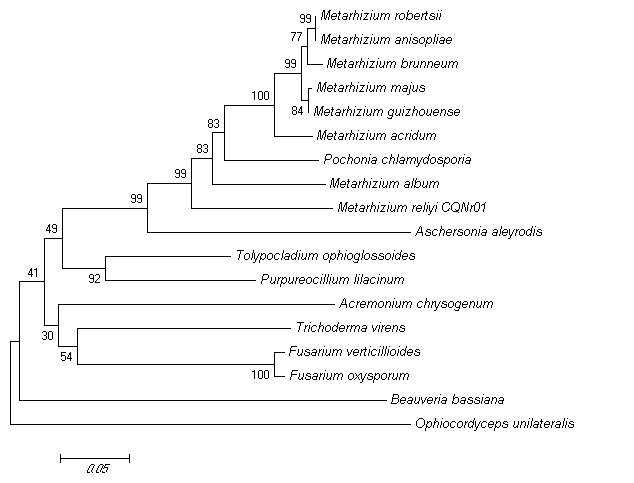


The numbers at the nodes represent the results of bootstrap analyses (1000 replicates) carried out using the neighbor-joining method. The sequences aligned with the MrMsn2 of *M. rileyi* are from *Metarhizium robertsii* ARSEF 23 (XP_007821913.1); *Metarhizium anisopliae* (KFG81865.1); *Metarhizium guizhouense* ARSEF 977 (KID91163.1); *Metarhizium majus* ARSEF 297 (XP_014578893.1); *Metarhizium brunneum* ARSEF 3297 (XP_014549267.1); *Metarhizium acridum* CQMa 102 (XP_007807348.1); *Metarhizium album* ARSEF 1941 (KHN96274.1); *Pochonia chlamydosporia* 170 (XP_022284582.1); *Aschersonia aleyrodis* RCEF 2490 (OAA32678.1); *Tolypocladium ophioglossoides* CBS 100239 (KND94659.1); *Purpureocillium lilacinum* (OAQ84437.1); *Fusarium oxysporum* Fo47 (EWZ44552.1); *Fusarium verticillioides* 7600 (XP_018749981.1); *Trichoderma virens* Gv29-8 (XP_013949616.1); *Beauveria bassiana* D1-5 (KGQ12306.1); *Acremonium chrysogenum* ATCC 11550 (KFH47964.1); *Ophiocordyceps unilateralis* (KOM17872.1).

**Fig. S2** Confirmation of gene disruption and complementation


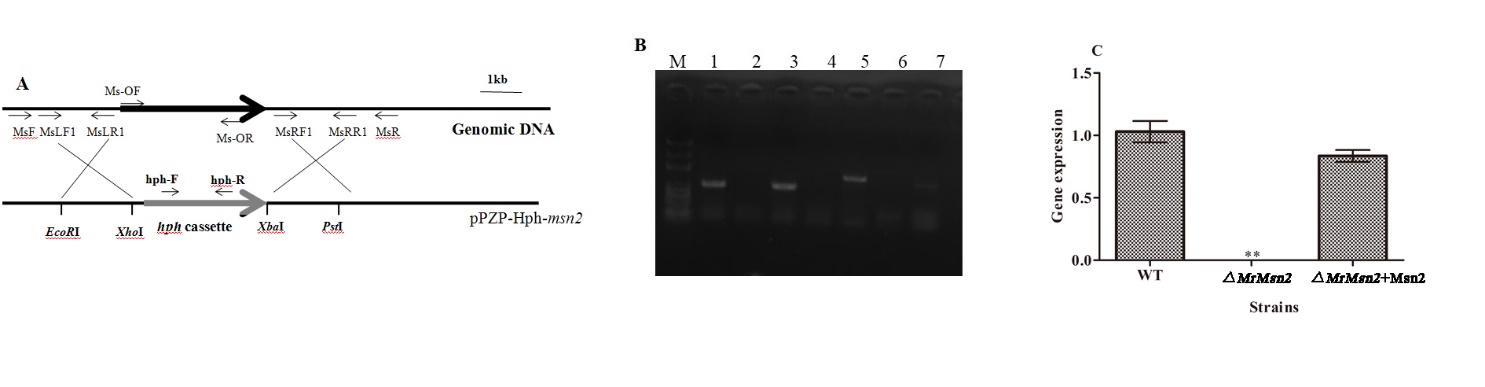
(A) The construct of *MrMsn2* loci, disruption constructs with *hph* cassette and flanking sequences. Arrows indicate the positions of primers used for PCR screening of deleted mutants. (B) PCR characterization of *△MrMsn2* mutants, wild-type, and CP strains. Open reading frame (ORF) was PCR using primers Ms-OF/Ms-OR (Lanes 1-3: 1-*MrMsn2* of wild-type (WT), 2-*△MrMsn2*, 3-*△MrMsn2+*Msn2); The *hph* and genomic sequence outside the flank regions was PCR using primers *hph*-F/*hph*-R and MsF/MsR (Lanes 4-7: 4-Left Frame (LF)-wild-type, 5-LF-*△MrMsn2*, 6-Right Frame (RF)-wild-type, 7-RF-*△MrMsn2*). M, DNA molecular size markers (DL 5000, Takara, Beijing). (C) RT-qPCR analysis for the expression analysis of *MrMsn2* in the different strains. Error bars represent ± SE.* *P*< 0.05, ** *P*< 0.01, significantly different when compared with the results obtained for the wile-type strain in AM cultures.

**Fig. S3** Morphology analysis and conidial yield of Morphology analysis and conidial yield of wild-type (WT), *△MrSwi6* mutants and complemented (CP) strains mutants under abiotic stress


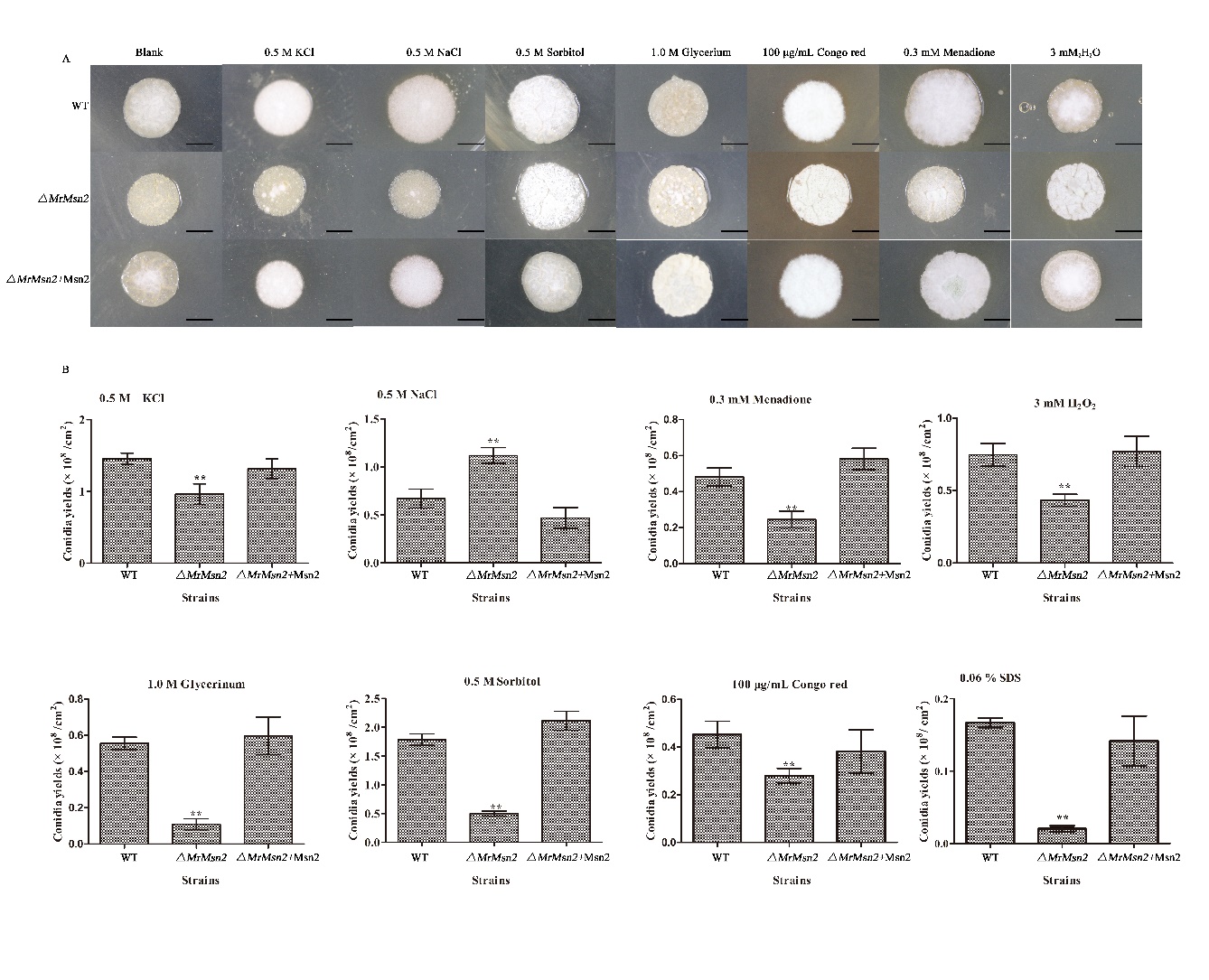


(A) Clone morphology of *M rileyi* WT, CP, and *△MrMsn2* mutants grown on SMAY with abiotic stress at 3 days. 3 μL conidial suspension (1× 10^7^ conidia) of tested strains were pipetted on SMAY plates with abiotic stress material (0.5 M KCl, 0.5 M sorbitol, 1.0 M glycerium, 0.06 % SDS, 100 μg/mL Congo red, 0.3 mM menadione, or 3 mM H_2_O_2_) under continuous light at 28 °C for 12 days. Bar=5 mm. (B) Conidial yield after 12 days of incubation. Error bars are standard error. * *P*< 0.05, ** *P*< 0.01 compared with wild-type.
